# Hunting, Food Preparation, and Consumption of Rodents in Lao PDR

**DOI:** 10.1371/journal.pone.0133150

**Published:** 2015-07-21

**Authors:** Kanokwan Suwannarong, Robert S. Chapman, Cecile Lantican, Tula Michaelides, Susan Zimicki

**Affiliations:** 1 FHI 360, Asia-Pacific Regional Office (APRO), Bangkok, Thailand; 2 College of Public Health Sciences, Chulalongkorn University, Bangkok, Thailand; 3 FHI 360, Lao PDR Office, Vientiane, Lao PDR; 4 FHI 360, Washington DC Office, Washington DC, United States of America; Royal Tropical Institute, NETHERLANDS

## Abstract

A cross-sectional study was conducted in 29 villages of Khamkeuth District in Bolikhamxay Province in the Lao PDR during March to May 2013. The study aimed to determine the characteristics associated with rodent consumption and related behaviors among different ethnic groups, ages, and genders. Five-hundred-eighty-four (584) males and females from 18-50 years of age participated in this study. Half of them were Hmong (292, 50%) while 152 respondents were Lao-Tai (26%) or other ethnic groups (140, 24%). Most of the respondents (79.5%) had farming as their main occupation. Prevalences of the studied outcomes were high: 39.9 for hunting or capturing rodents in the previous year, 77.7% for preparing rodents as food, and 86.3% for rodent consumption. Multivariable logistic regression analysis showed that likelihood of these types of rodent contact was more consistently associated with behavioral factors (gathering things from the forest and elsewhere, cultivation-related activities, and taking measures to prevent rodent-borne disease) than with socio-demographic, environmental, or cultural factors. The strongest associations were observed for gathering things; these associations were consistently positive and statistically significant. Although this study did not directly assess rodent-borne zoonosis risk, we believe that study findings raise concern that such risk may be substantial in the study area and other similar areas. Further epidemiological studies on the association between rodent-borne disease infection and rodent hunting, preparation for food, and consumption are recommended. Moreover, further studies are needed on the association between these potential exposure factors (i.e., rodent hunting, preparation for food, and consumption) and rodent-borne infections, especially among ethnic groups like the Hmong in Lao PDR and those in neighboring countries with similar socio-demographic, environmental, behavioral and cultural contexts.

## Introduction

The United States Agency for International Development (USAID) launched the global Emerging Pandemic Threats (EPT) program in 2009 to combat emerging infectious diseases that could be harmful to humans. PREVENT was one of the four projects under the EPT program, and focused on using behavior change and communication strategies to prepare for, prevent, respond to, and control pandemic influenza and other emerging threats. Specifically, PREVENT aimed to identify key populations at highest potential risk of exposure to emerging pathogens based their interactions with domestic animals and wildlife. PREVENT launched several research activities in Africa and Southeast Asian countries such as Cambodia, Lao PDR, Thailand and Vietnam. One of the PREVENT’s significant human-animal interface research studies in Southeast Asia was conducted in Lao PDR. This, like other studies in PREVENT, was intended to quantify, and identify factors associated with, human exposure to animals. This would assist in identifying populations potentially at increased risk of contracting an emerging infectious disease. These results are expected to inform the development of interventions that could reduce this risk.

In 2010, the population of Lao PDR was 6.2 million [1], with an estimated annual growth rate of 1.7%. As of the early 2000s it was estimated that 80% of the population was living in rural areas [2] and in 2007/2008, 71% was engaged in agriculture [3]. The government recognizes over 200 linguistic groups belonging to 49 distinct ethnic populations [3]. In the past, Lao PDR’s ethnic diversity was commonly aggregated under three main groups [4,5].

Based on a desk review [6] of the published literature, sources revealed that Lao Loum (Lao from the lowlands), comprised mainly of Lao and Tai populations, lived in lowland areas, was predominantly involved in wet rice cultivation, and also engaged in animal husbandry and fishing. Their population was estimated to make up 68% of the total Lao population in 2002. Lao Theung (Lao from the mid-range-highlands), comprised mainly of Mon-Khmer populations, lived in highland areas, grew upland (dry) rice, and relied strongly on other agro-forestry activities (fishing, hunting, collecting forest products). They were estimated to make up 22% of the total population in 2002.Lao Sung (Lao from the uplands), were comprised of mostly Hmong and Yao populations that traditionally lived on subsistence agriculture based on slash-and-burn practices and occupied areas higher than the Lao Theung (above 1,000 m.a.s.l.). They were estimated to make up 10% of the total population in 2002. This “classification” does not have an anthropological basis and has been officially abandoned. It is, however, still widely used in reports.

The PREVENT project addresses a wide variety of animals. The current report focuses on human contact with rodents, which are an important subset of these. Rodents ***(rodentia order)*** are mammals characterized by two continuously growing incisors in the upper and lower jaw, which must be kept short by gnawing. Rodents are of special interest under the EPT Program because they have become an increasingly significant health risk worldwide, especially in Southeast Asia [7–11]. Important rodent-borne zoonotic infections include leptospirosis [12–19], hantavirus infection [9,20–22], arena viruses [23], scrub typhus [24–26], Plague [7,27–30], parasitic diseases [11,31], toxoplasmosis [32] and food poisoning [33].

As of 2007, 53 types of rodents [34] had been identified in the Lao PDR. According to Duckworth, Salter and Khounboline [35], common rodents in Lao PDR in 1999 included:

***Muridae*** (rats and mice): murinae sub-family, between 28 and 31 species are found in Lao PDR
***Rhyzomyinae*** (bamboo rats): three species are found in Lao PDR
***Arvicolinae*** (voles): between zero and two species are potentially found in Lao PDR
***Soricidae*** (shrews): five, potentially seven species are found in Lao PDR (of 272 species worldwide)
***Sciuridae*** (non-flying squirrels): nine to 12 species are found in Lao PDR (out of 230 worldwide)
***Pteromyidae*** (flying squirrels): six to eight species are found in Lao PDR (among 43 worldwide)
***Hystricidae*** (porcupines): two species are found in Lao PDR (among 11 worldwide)

Hunting and eating of wildlife by man is an ancient practice that carries a substantial risk for zoonotic disease transmission [36]. The practice is still of global importance because of increases in human population density, globalized trade, and the consequent increased contact between humans and animals [36]. Hunting activities of animals including rats is common [37,38]. Butchering (opening, cutting, dressing, and preparing the carcass) poses higher risk for blood-borne pathogens than does the transportation, sale, purchase, and consumption of the butchered meat [36]. Consuming wild foods is part of the culture in many societies, including farming populations [39]. Consumption of rodents, especially rats and squirrels, is quite popular in Asia [40]. Rodents are also used for feasts, religious ceremonies, and in exchange for certain activities [41]. Knowledge of non-domesticated food resources is part of traditional and tacit ecological knowledge. The cultural knowledge of wild food resources and the practices of hunting and gathering have been transmitted from generation to generation [39]. A study found that 62% of household food was made up of wild food resources (31%came from the forest and 31%came from paddy field), 22% was produced by the household, 13% was purchased, and 3% were gifts [42].

Because rodents are significant reservoirs for several zoonoses, hunting and preparing rodents as food, as well as and rodent consumption, could be important factors in the transmission of zoonotic infections [43]. To date, there have been very few studies on zoonoses in the Southeast Asian region [9], especially as related to rodent consumption [8,44]. Therefore, this study was conducted and focused on rodent-human interactions during rodent hunting, preparation as food, and consumption among residents in Bolikhamxay Province of the Lao PDR. These hope was that this study would provide information on the potential risk of zoonotic diseases associated with rodent hunting, preparation, and consumption in different ethnic groups in the country. Results should be useful in characterizing zoonosis exposure risk based on activities among specific ethnicities, and perhaps can be generalized to apply to populations elsewhere in Lao PDR, as well as in neighboring countries with similar socio-demographic, environmental, behavioral and cultural contexts.

## Materials and Methods

### Study Design, Study Area, and Study Sites

This cross-sectional study was conducted during March-May 2013 in villages in Khamkeuth District, Bolikhamxay Province, located in the central part of Lao PDR (S1 Fig). The province was selected in consultation with the Lao PDR government, represented by the Department of Hygiene and Prevention, Ministry of Health (MOH), the National Emerging Infectious Diseases Coordination Office (NEIDCO), the Department of Livestock and Fisheries, and the Wildlife Division of the Ministry of Agriculture and Forestry (MAF).

This province hosts several dams and a protected forest area that is home to both wild animal and human communities that subsist on hunting. Deforestation of tropical forest areas is one cause of increasing contact between wildlife and hunters [36]. Therefore, it was important to select a site where the environment is changing due to development-related human activities (e.g., logging, construction of dams, and presence of other extractive industries) that can transform the human-animal interface in ways that could potentially result in new exposures to disease. Ultimately, the study site would have people likely to have a high level of contact with animals that could be reservoirs of emerging infections, and where the human population is sufficiently dense and/or sufficiently mobile to spread the infection.

### Respondents and Household Selection

Respondents were selected according to the following criteria:

#### Ethnicity

The two main ethnic groups, Hmong and Lao-Tai, who were living in Khamkeuth District, were selected for inclusion in the study. The inclusion criteria were the ethnic residents, aged between 18–50 years old and lived in the study areas at least 6 months before data collection.

The selection of different ethnic groups presented a logistical challenge, as it involved work in multiple languages. It also provided an opportunity for examining how culture may affect contact with animals, and for identifying socially determined factors potentially associated with rodent-borne zoonotic infections.

The Lao-Tai ethnic group is the largest and culturally dominant group in the country; their language (Lao) is the official language in Lao PDR. The Lao-Tai, belonging to the same language group as the Lao, are closely related both culturally and linguistically, sharing 90% of their vocabulary. The Lao and Lao-Tai mostly consider themselves Buddhist. The Hmong, the third-largest group in Lao PDR, are still considered an ethnic minority, as they comprise less than 10% of the population. The Hmong originally migrated from China in the 18th century, and their language is derived from Chinese. Some Hmong have converted to Christianity, but most still practice some form of ancestral worship and shamanism. While they traditionally practice slash-and-burn agriculture, the Hmong now also harvest different forest products, including wild animals, to sell.

#### Age and Gender

In each of the four study communities which included two each of Hmong and Lao-Tai communities, household interviews were conducted among adult females and males 18–50 years of age. These respondents were selected to assess the effect of gender and age on exposure to animals. It was hypothesized that men and women have different types or rates of exposure related to specific gender roles in the society.

A two-stage cluster sampling procedure was used. In the first stage, villages were selected randomly using probability proportional to size (PPS) sampling. In the second stage, independent samples of males and females in households in each village were selected using systematic sampling with different random starts for the two genders, and with a specified interval between selected households. Each team used a predetermined walking route that covered the entire village so that all households in the villages had an equal chance of being included in the survey. This route was determined prior to the start of fieldwork using GIS points provided by GPS machines. Starting with the first household of each sample and walking in the predetermined route, the survey team screened for eligible respondents in households. In households with more than one eligible adult, one adult was selected by using a Kish grid table [45], which essentially gave an equal probability of selection to each eligible respondent in the village.

### Data Collection Tools and Procedures

The study used a standardized questionnaire, consisting of 13 sections, which elicited information on socio-demographic and other descriptive characteristics, and evidence of contact with rodents and other animals, (including poultry and domestic animals). As mentioned above, this report focuses specifically on rodents. After the original questionnaire was translated from English to Lao and Hmong, a pre-test was conducted to gauge the validity and precision of the translation, as well as clarity of the questions. The pre-test was conducted among 40 individuals (21 Hmong and 19 Lao-Tai. In light of pre-test findings, questionnaires in English, Lao, and Hmong were refined before they were used in full-scale data collection. Interviews were conducted by trained field researchers.

### Study Variables

#### Dependent Variables

Three outcomes were studied, as follows.

#### Respondents reported hunting or capturing rodents in the past year

This outcome variable came from questions that asked whether the respondents reported hunting or capturing animals, including rodents, in the past year.

#### Respondents reported preparing rodents for food

Questions for this outcome variable asked whether the respondents prepared animals, including rodents, for food by slaughtering, butchering, and/or cutting up rodents in the past year.

#### Respondents reported eating rodents in the past year

This outcome variable was from a question that asked whether the respondents reported any consumption of animals, including rodents, in their families during the year before data collection occurred.

#### Independent Variables

Twenty-two independent variables were considered in the analysis. These were based on unpublished formative research in Khon Kaen Province of Thailand by PREVENT during 2011 [46] and literature which addressed factors related to rodent consumption and hunting [37], such as socio-demographic factors (e.g., age, gender, occupation, and economic status) [39,47–49], behavioral factors (e.g., cultivation-related tasks) and environmental factors (e.g., household types) [50]. This research indicated that potential factors such as age, gender, economic status, and cultural context might be associated with rodent exposure. Likewise, previous research [50]showed several environmental factors (e.g., household types) were associated with rodent contact and rodent-borne disease infections (e.g., hantavirus). Of these, 18 were dichotomous, 1 was categorical (3 groups), and 3 were continuous. The independent variables were grouped into four types: socio-demographic, environmental, behavioral, and cultural. Independent variables are listed below, showing comparison groups vs. reference groups.

### Socio-demographic Information

Age group (>36 vs. ≤36 years)

Gender (male vs. female)

Ethnicity (Lao-Tai, Hmong, and other (ref))

Religion (spirit vs. other)

Education attainment level (≥secondary school vs. other)

Marital status (married or cohabiting vs. other)

Types of occupation (farmer vs. other)

Family size (>6 vs.≤6 people)

Has a car (yes vs. no)

### Environmental Information

Sanitation types (flush toilet vs. other)

Main drinking water source (using rainwater in all seasons vs. other)

Animals have access to drinking water (yes vs. no)

Waste disposal (waste collected vs. other)

Main cooking fuel (biomass vs. other)

Dwelling has wooden floor (yes vs. no)

Dwelling has wooden walls (yes vs. no)

Dwelling has zinc roof (yes vs. no)

### Behavioral Information

Number of food crops grown (continuous)

Number of things gathered from the forest or other places, e.g., fruits, vegetables, insects, animal waste, wood (continuous)

Takes measures to avoid rodent-borne disease (yes vs. no)

### Cultural Context

Knowledge/attitude toward animal-borne disease (continuous score)

Aware that rodents can cause human disease (yes vs. no)

### Statistical Analysis

Data were analyzed separately for the three outcome variables and included all 584 subjects. During analysis, descriptive statistics were calculated for dependent and independent variables. Then data were analyzed in three steps. Step 1 consisted of bivariate analysis in which associations between the dependent variables and each of the independent variables, considered separately, were ascertained [51]. All three dependent variables were dichotomous. Thus, chi-square or Fisher's exact tests were used for bivariate analyses, and logistic regression was used for subsequent analyses, as described below.

In Step 2, a multiple logistic regression model, which included all independent variables for which p≤0.15 in the bivariate analysis, was constructed for each dependent variable. In Step 3, a second logistic regression model, which included independent variables for which p≤0.15 in the Step 2 model, was constructed for each dependent variable. P-values ≤0.05 were considered statistically significant. Data analysis was conducted with SPSS software (version 22; IBM: Armonk, NY).

### Ethical Considerations

This study was conducted after obtaining ethical approval from the FHI 360 Institutional Review Board (IRB), the Lao PDR National Ethics Committee for Health Research (NECHR) within the National Institute of Public Health (NIOPH) under the Ministry of Health, and the College of Public Health Sciences, Chulalongkorn University.

This study included no invasive or medical procedures of any kind. Participation in the study was strictly voluntary. Written informed consent was obtained from all respondents before proceeding to interview/discussion. Participants were assured that their responses were not shared by the researchers and were kept completely confidential and private. They were provided information about whom to contact if they had questions about the study. Measures were taken to ensure the respect, dignity, and freedom of each participant. During training of fieldworkers, obtaining informed consent, avoiding coercion of any kind, and maintaining confidentiality was emphasized. To the extent possible, the interviews were conducted in a private setting where the interviews could not be heard by others.

## Results

Five hundred and eighty-four (584) respondents from 29 villages of Khamkeuth District in Bolikhamxay Province participated in this study. The selection of each village was based on 2011 census information on the location of the main two ethnicity groups (Lao-Tai and Hmong). Among all respondents, half of them were Hmong (292, 50%) while 152 respondents were Lao-Tai (26%) and 140 (24%) were of other ethnic groups. Most of them (79.5%) said their main occupation was farmer, followed by housewife (12.0%) and trader (4.3%). The mean duration of living at the location of their interview was 20.52 years. The minimum and maximum periods of living in that location were 1 and 47 years, respectively.

Results of the three outcomes were as follows:

### Respondents reported hunting or capturing rodents in the past year

Two hundred and thirty-three (39.9%) respondents (30 females and 203 males) reported hunting or capturing rodents in the past year. Their mean age was 32.9 years old and 85 of the respondents (36.5%) were >36 years old. Most of them were married or cohabitating (216, 92.7%) and their main occupation was farmer (210, 90.1%). Ninety-one of the respondents (39.0%) had higher than primary school education, and 114 respondents (48.9%) lived in households with > 6 people.

The respondents reported hunting or capturing specific rodent species in the past year, namely: Chipmunks (2, 0.9%), Porcupines (14, 0.6%), Rats/mice (187, 80.3%), and Squirrels (140, 60.1%).

Bivariate analysis showed that nine independent variables (male gender, educational attainment level, farmer occupation, has a car, main drinking water as opened natural resources, animals have access to drinking water, take measures to avoid rodent-borne diseases, number of food crops grown, and number of things gathered) were associated with this dependent variable, at p≤0.15. These were entered in the initial multiple logistic regression model, which is summarized in Table 1. Five independent variables (male gender, farmer occupation, has a car, take measures to avoid rodent-borne diseases, and number of things gathered) were eligible for the step 2 analysis, which is summarized in Table 1.

**Table 1:** First multiple logistic regression model for reporting hunting or capturing rodents in the past year.

Variables	Coefficient	Odds Ratio (95% CI)	P-values
Male gender	3.192	24.347 (14.600–40.601)	<0.001
Educational attainment	0.220	1.246 (0.749–2.073)	0.397
Farmer occupation	1.034	2.812 (1.473–5.370)	0.002
Has a car	-0.438	0.645 (0.374–1.115)	0.116
Main drinking water from open natural resources	0.118	1.125 (0.704–1.797)	0.622
Animals have access to drinking water	0.062	1.064 (0.614–1.846)	0.824
Take measures to avoid rodent-borne diseases	-0.377	0.686 (0.435–1.082)	0.105
Number of food crops grown	0.085	1.088 (0.846–1.400)	0.511
Number of things gathered	0.493	1.638 (1.342–1.999)	<0.001
Constant	-4.413	0.012	<0.001

Table 2 shows that three independent variables were positively and significantly associated with hunting or capturing rodents in the past year. These were male gender (OR = 25.719, 95% CI 15.576–42.466, p<0.001), farmer occupation (OR = 2.740, 95% CI 1.494–5.024, p<0.001), and number of things gathered (OR = 1.656, 95% CI 1.361–2.016, p<0.001). Having a car and taking measures to avoid rodent-borne diseases were no significantly associated with this outcome.

**Table 2:** Second multiple logistic regression model for reporting hunting or capturing rodents in the past year.

Variables	Coefficient	Odds Ratio (95% CI)	P-values
Male gender	3.247	25.719 (15.576–42.466)	<0.001
Farmer occupation	1.008	2.740 (1.494–5.024)	0.001
Has a car	-0.410	0.664 (0.386–1.141)	0.138
Take measures to avoid rodent-borne diseases	-0.378	0.685 (0.436–1.079)	0.103
Number of things gathered	0.505	1.656 (1.361–2.016)	<0.001
Constant	-4.079	0.017	<0.001

### Respondents reported preparing rodents in the past year

Four hundred and fifty-four (77.7%) respondents (233 females and 221 males) reported that they prepared rodents in the past year. The mean age was 32.3 years and 146 of the respondents (32.2%) were >36 years old. Most of them were married or cohabitating (414, 91.2%) and their main occupation was being a farmer (376, 82.2%). One hundred and forty of the respondents (30.8%) had higher than primary school education, and 228 respondents (50.2%) lived in households with >6 people.

Based on bivariate analysis results, nine independent variables (age, ethnicity, spirit religion, farmer occupation, family size, dwelling has wooden walls, takes measures to avoid rodent-borne diseases, number of cultivation-related tasks, and number of things gathered) were included in the initial logistic regression model. This step is summarized in Table 3.

**Table 3:** First multiple logistic regression model for reporting preparing rodents in the past year.

Variables	Coefficient	Odds Ratio (95% CI)	P-values
Age group	-0.710	0.492 (0.321–0.752)	0.001
Ethnic group			0.266
Ethnic group–Lao-Tai*	-0.225	0.798 (0.456–1.398)	0.431
Ethnic group—Hmong*	0.320	1.377 (0.730–2.598)	0.323
Religion	0.060	1.061 (0.601–1.874)	0.837
Farmer occupation	0.348	1.416 (0.848–2.365)	0.184
Family size	-0.174	0.840 (0.542–1.303)	0.437
Dwelling with wooden walls	0.175	1.191 (0.776–1.829)	0.423
Take measures to avoid rodent-borne diseases	0.313	1.367 (0.889–2.104)	0.155
Number of cultivation-related tasks	0.098	1.103 (0.873–1.394)	0.412
Number of things gathered	0.377	1.457 (1.212–1.752)	<0.001
Constant	-0.119	0.888	0.807

* As compared to ethnic group = other.

Only two independent variables (age group and number things gathered) were eligible for the step 2 of analysis. Table 4 shows that both of these variables were significantly associated with preparing rodents in the past year. This outcome was positively associated with number of things gathered (OR = 1.519, 95% CI 1.280–1.802, p<0.001), and negatively associated with being >36 years old (OR = 0.469, 95% CI 0.313–0.704, p<0.001).

**Table 4:** Second multiple logistic regression model for reporting prepared rodents in the past year.

Variables	Coefficient	Odds Ratio (95% CI)	P-values
Age group	-0.757	0.469 (0.313–0.704)	<0.001
Number of things gathered	0.418	1.519 (1.280–1.802)	<0.001
Constant	0.617	1.854	0.007

### Respondents reported eating rodents in the past year

Five hundred and four (86.3%) respondents (250 females and 254 males) reported that they had eaten rodents in the past year. Their mean age was 33.1 years old and 185 respondents (36.7%) were >36 years old. Most of them were married or cohabitating (462, 91.0%) and their main occupation was farmer (263, 91.7%). One hundred and fifty-eight respondents (31.4%) had higher than primary school education, and about half of the respondents (253, 50.2%) lived in households with >6 people.

Three hundred and ninety-eight respondents (79.0%) reported that they had eaten rat/mouse over the past year while 366 respondents (72.6%) reported having eaten squirrels, 46 respondents (9.1%) reported having eaten porcupines, and 10 respondents (2.1%) reported having eaten porcupines in the past year.

Based on bivariate analysis results, eight independent variables (ethnicity, spirit religion, farmer occupation, family size, dwelling with wooden walls, take measure to avoid rodent-borne diseases, number of cultivation-related tasks, and number of things gathered) were included in the initial logistic regression model (this is summarized in Table 5). Four independent variables (ethnicity, take measure to avoid rodent-borne diseases, number of food crops grown, and number of things gathered) were eligible for the Step 2 of the analysis (Table 6).

**Table 5:** First multiple logistic regression model for reporting eaten rodents in the past year.

Variables	Coefficient	Odds Ratio (95% CI)	P-values
Ethnic group			0.005
Ethnic group–Lao-Tai*	-0.984	0.374 (0.184–0.758)	0.006
Ethnic group—Hmong*	0.093	1.097 (0.487–2.472)	0.823
Sprit religion	-0.100	0.905 (0.450–1.819)	0.779
Farmer occupation	0.391	1.478 (0.814–2.684)	0.199
Family size	-0.327	0.721 (0.421–1.234)	0.233
Dwelling with wooden walls	0.161	1.175 (0.694–1.988)	0.548
Take measure to avoid rodent-borne diseases	0.394	1.483 (0.888–2.478)	0.132
Number of cultivation-related tasks	0.232	1.261 (0.955–1.666)	0.102
Number of things gathered	0.389	1.475 (1.184–1.839)	0.001
Constant	0.356	1.427	0.531

* As compared to ethnic group = other.

**Table 6:** Second multiple logistic regression model for reporting eating rodents in the past year.

Variables	Coefficient	Odds Ratio (95% CI)	P-values
Ethnic group			<0.001
Ethnic group–Lao-Tai*	-0.991	0.371 (0.187–0.736)	0.005
Ethnic group—Hmong*	0.208	1.231 (0.612–2.475)	0.560
Takes measures to avoid rodent-borne diseases	0.396	1.486 (0.895–2.464)	0.125
Number of cultivation-related tasks	0.308	1.361 (1.042–1.777)	0.023
Number of things gathered	0.426	1.532 (1.238–1.895)	<0.001
Constant	0.163	1.177	0.740

* As compared to ethnic group = other.

Table 6 shows that the likelihood of eating rodents in the past year was considerably lower in the Lao-Tai ethnic group than in other groups. Also, rodent consumption was positively and significantly associated with the number of things gathered (OR = 1.532, 95% CI 1.238–1.895, p< 0.001), and with the number of cultivation-related tasks (OR = 1.361, 95% CI 1.042–1.777, p 0.023).

## Discussion and Conclusion

The present study did not directly characterize zoonosis risk in relation to the types of rodent contact assessed. Also, very little is known regarding risk of rodent-borne zoonoses in the study area and elsewhere in the Lao PDR. Even so, for reasons given below, we believe that the present findings raise substantial concern regarding potential risk of rodent-borne zoonoses in the study area. First, many respondents reported rodent contact via hunting, preparing, or eating. Prevalences of the studied outcomes ranged from 39.9% for hunting rodents to 86.3% for consuming them. Indeed 533 respondents (91.3%) were positive for at least one outcome. Second, rodent consumption and related activities have been associated with zoonosis risk in locations other than the study area [41, 49].

Third, in study respondents, behavioral factors (gathering, growing crops, and taking measures to avoid rodent-borne disease) were considerably more consistently associated with the studied outcomes than were socio-demographic, environmental, or cultural factors. Among behavioral factors, the strongest and most consistent associations were observed for gathering things in the forest and elsewhere; the OR per unit increase in number of things gathered ranged from 1.52 for preparing rodents to 1.65 for hunting rodents (P<0.001 for all outcomes). These translate to very large ORs for the outcomes of gathering 5 things, compared to gathering no things. These ORs ranged from 8.08 for preparing rodents to 12.47 for hunting them. Also, unadjusted prevalences of all outcomes increased monotonically and significantly (p<0.001) as the total number of things gathered increased from 0 to 5. Specifically, these prevalences ranged from 16.7% to 76.9% for hunting, from 55.6% to 96.2% for preparing, and from 61.1% to 100.0% for consuming rodents. Furthermore, a large proportion of respondents (276 or 47.3%) reported gathering 3 or more things in the past year. In view of the observed high prevalence of gathering, coupled with the high relative and absolute risks of rodent contact associated with it, we believe that the study population is at substantial risk of rodent-borne zoonoses via rodent consumption and related activities. Further research is of course required to address this question directly.

The present results were generally consistent with several other studies, including findings of an unpublished PREVENT formative study [46] which found that males had a greater chance to interact with wild animals than females did. A study in Khon Kaen Province in 2011 [52] also revealed that males was associated with reported rodent consumption; this might be the case because males were the main family members who worked crops and were thus at greater risk of exposure to rodents and also more likely to hunt, prepare, and eat them. A World Wildlife Fund survey carried out in *Phrai* communities in Xayabury province confirms that villagers hunt predominantly in hills and rice fields rather than in the forest [53].

In 1999, Duckworth, Salter and Khounboline [35] summarized the findings of village interviews conducted between 1988 and 1993 throughout Lao PDR pertaining to wildlife consumption. Out of a panel of 317 interviewees, 24.6% reported that squirrels are among the three most common wild meats eaten. Civets were mentioned by 21.8% of the interviewees, primates by 12.6%, rodents by 8.8%, and porcupines by 5.7% of the interviewees. A survey carried out in seven villages in the Nam Et-PhouLoey NPA in 2001 [5] revealed the following ranking in terms of most hunted animals: 1) squirrels, 2) red jungle fowl, 3) pheasants, 4) common barking deer, and 5) wild pigs. The same author, however, noticed substantial differences across villages, even within the same ethnic community. Some may find badgers and leopard cats a delicacy, while others prefer squirrels and jungle fowl [5].

In conclusion, these findings lead us to recommend that further epidemiological studies be conducted on potential exposure factors (such as hunting, preparing rodents for food, and rodent consumption) that have a direct association with rodent-borne infections, especially among ethnic groups like the Hmong in Lao PDR and in neighboring countries with similar socio-demographic, environmental, behavioral and cultural factors as Lao PDR. As mentioned above, direct characterization of zoonosis risk in relation to the studied types of rodent contact is also needed.

## Supporting Information

S1 Fig Maps of Bolikhamxay Province, Lao PDR. (TIF)

S1 File Compressed/ZIP File Archive. (ZIP)
