# Supplementary figures and images for: Hunting, Food Preparation, and Consumption of Rodents in Lao PDR
Source: PLoS One. 2015 Jul 21;10(7):e0133150. doi: 10.1371/journal.pone.0133150 (PMC4511002; doi:10.1371/journal.pone.0133150)

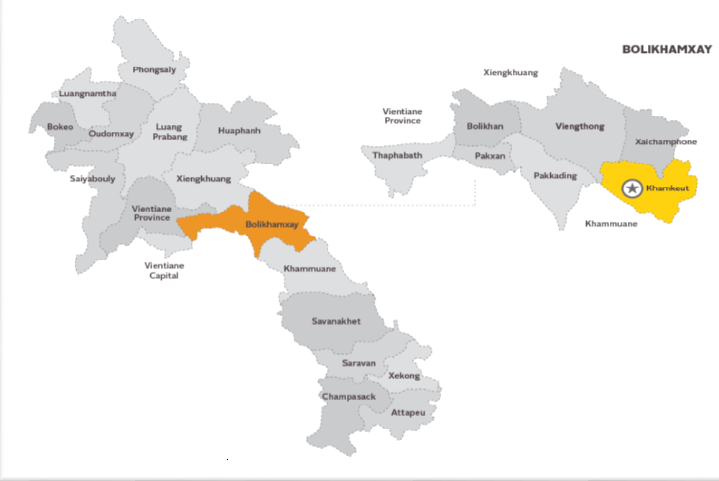

Supplement: S1 Fig — (TIF) [file pone.0133150.s001.tif]
